# Fluorescent Ionic Probe for Determination of Mechanical Properties of Healed Poly(ethylene-*co*-methacrylic acid) Ionomer Films

**DOI:** 10.1021/acsapm.1c01325

**Published:** 2022-02-02

**Authors:** Caitlan E. Ayala, Rocío L. Pérez, John K. Mathaga, Aanesa Watson, Tristan Evans, Isiah M. Warner

**Affiliations:** † Department of Chemistry, Louisiana State University, Baton Rouge, Louisiana 70803, United States; ‡ Department of Chemistry and Biochemistry, Georgia Southern University, Statesboro, Georgia 30458, United States; § Department of Chemistry, Fort Valley State University, Fort Valley, Georgia 31030, United States

**Keywords:** fluorescent probe, ionic materials, GUMBOS, ionomer self-healing, ratiometric sensing, recovered mechanical properties

## Abstract

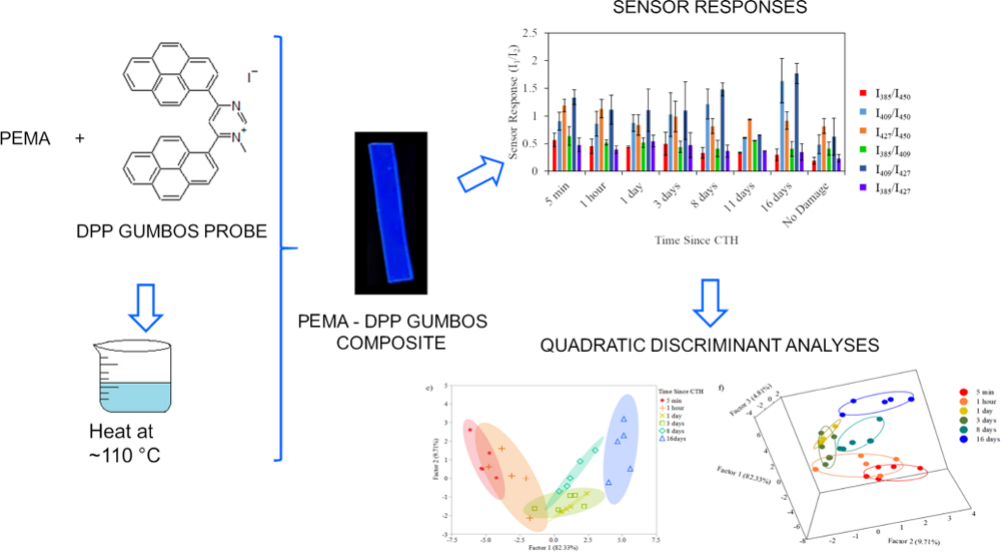

In recent years, advanced materials with properties resembling biological systems, particularly artificial muscles, have received intense scrutiny. This is because the interesting conformational shape characteristics of such materials have benefited a variety of technologies, including textiles, 3D printing, and medical devices. Although a multitude of shape memory properties have been studied and developed in recent years, self-healing of these polymers after puncture or rupture has also become a major area of study. Most techniques for detection of such processes are mechanically based and require considerable hands-on monitoring. Thus, a rapid visual detection method for self-healing is highly desirable. Herein, we describe fluorescence studies for rapid detection of self-healing properties of a partially neutralized sodium ionomer poly(ethylene-*co*-methacrylic acid) (PEMA). In this study, two different fluorophores, parent non-ionic 4,6-dipyrenylpyrimidine and ionic 4,6-dipyrenylpyrimidinium iodide fluorophores, were evaluated as possible sensors of self-healing. Incorporation of these probes via solution blending and compatibility into a PEMA of these fluorophores were evaluated. Thermal characterizations using differential scanning calorimetry were also performed to elucidate physical characteristics of healed sites. Ratiometric fluorescence emission variations were explored within puncture-healed ionomer films and related to Young’s modulus properties with good linearity, indicating potential utility of this approach for monitoring elastic modulus properties after healing has occurred. Further statistical analyses of mechanical processes using quadratic discriminant analysis resulted in development of several highly accurate predictive models for determining time since damage healing.

## Introduction

Scientists strongly desire to improve the lifetimes of polymeric materials through ability of pre-defined systems to heal upon introduction of an external stimulus, that is, self-heal. For example, among other properties, recovery after crack or impact damage is of particular interest in fields such as aerospace, automotive, biotechnological, and industrial engineering.^1−4^ Several research groups have explored the ability to recover mechanical properties after damage healing of both microscopic and macroscopic crack closures within a multitude of materials.^5−7^ These damage recovery processes are known to undergo healing processes through a close-then-heal (CTH) mechanism.^8−14^ After a damage event in polymers, the damage healing mechanism occurs through constrained shape recovery to close cracks, followed by healing on a molecular scale. In recent years, ionic clusters as healing agents have been found to introduce this CTH capability through water-, solvent-, temperature-, and/or stress-induced shape recovery.^15−21^

Ionomers are defined as a subclass of materials that contain less than or equal to a 15 mol % ionic content. The use of such an ionic content introduces hydrophilic clusters that can alter thermal and mechanical properties via insertion of heterogeneity within the polymer matrix.^22−26^ As a result, incorporation of ionic cluster polymers has increased fracture resistance, toughness, tensile strength, along with self-healing capabilities in various systems.^27−29^ Variants of ionomers, such as poly(ethylene-*co*-methacrylic acid) (PEMA), have also been employed for several applications, including food and cosmetic packaging^30,31^ and solar cell technologies.^32,33^ Recent studies have been published where ionomers are used as self-sensing and self-healing agents for fabrication of self-healing composites.^10,17,20,27,34,35^

To date, optomechanistic research of large-scale damage sensing has predominantly focused on incorporating mechanochromic compounds for strain sensing of neutral polymeric systems.^36−38^ Many studies ranging from use of neutral mechanochromic sensors that rely on aggregation-induced emission processes (AIE) and^38,39^ dynamic covalent bond alterations,^40−43^ among others,^44,45^ have been evaluated for sensing. Weder and coworkers have reported an encapsulated solvatochromic dye as a probe to provide optical information based on polarity changes from surrounding polymer damage.^46^ More recently, chromophores, such as AIE luminogens and aggregation-induced quenching fluorophores, have been co-polymerized to serve as sensors for damage and/or self-healing detection in polymeric materials.^47,48^

**Figure 1:**
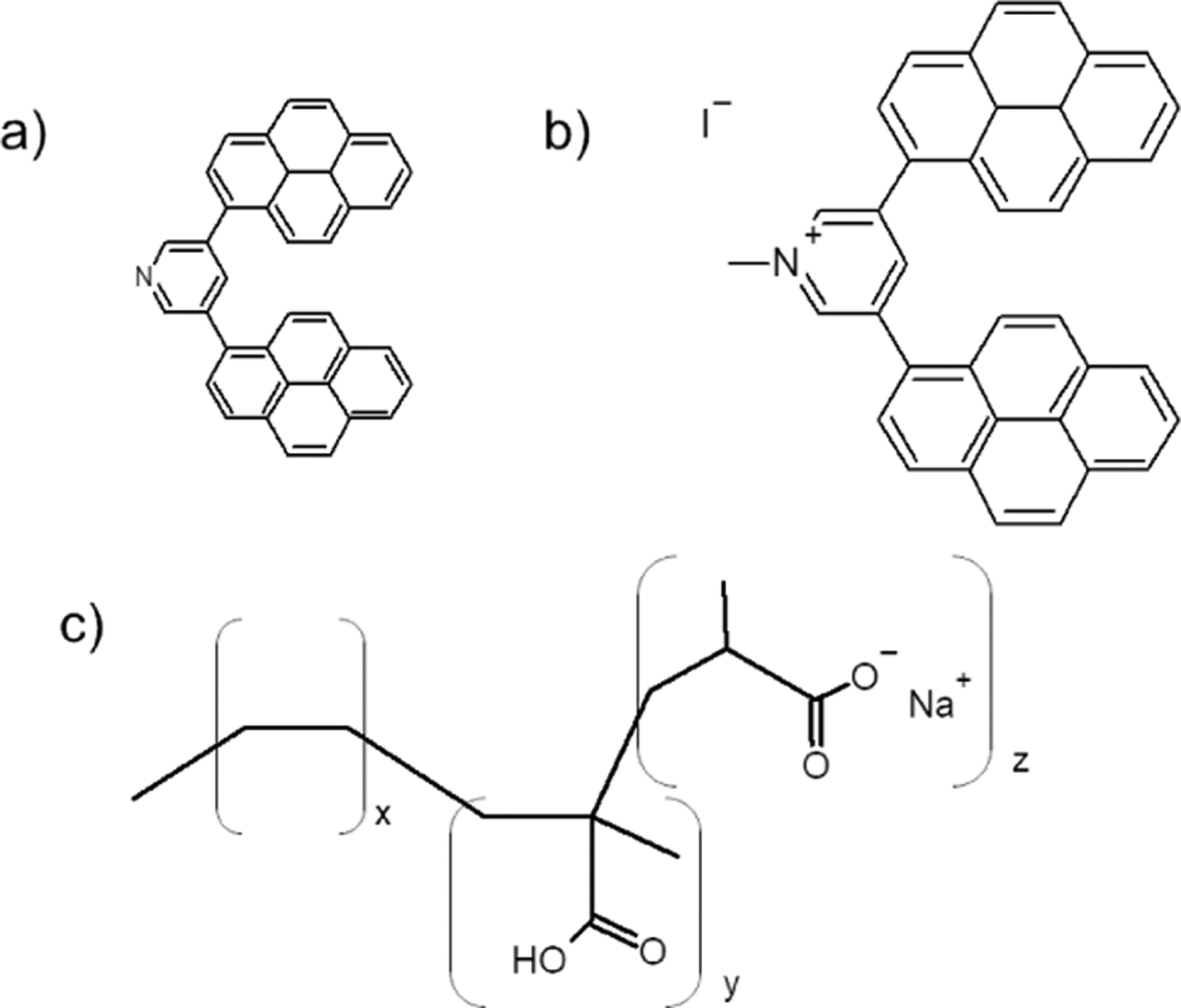
Structures of non-ionic fluorophores 4,6-dipyrenylpyrimidine (DPP) and 1-methyl-4,6-dipyrenylpyrimidinium iodide [(DPP)(I)].

Herein, we explore use of fluorophores containing pyrene subunits, a solvatochromic moiety,^49−52^ in a non-ionic precursor and a group of uniform materials based on organic salt (GUMBOS) derivative to explore (1) PEMA matrix compatibility via solution blending and (2) the ability to correlate damage-healing information with mechanical properties of the damaged site using optical methods. Structures of the fluorescent probes and ionomer matrix are shown in Figure 1. Similar in composition to ionic liquids, GUMBOS are solid-state ionic material analogues that have defined melting points ranging from 25 to 250 °C.^53^ Through strategic design of such ionic systems, these compounds create pathways to multi-functional materials with tunable functionalities in the solid state.^52,54−59^ In this regard, we hypothesize that since CTH in ionomers is predicated on induced shape recovery followed by localization of ionic species within the polymer network to restore mechanical properties,^46^ hydrophobic ionic fluorescent materials may prove useful as probes for optically elucidating mechanical properties after healing events.

## Results and Discussion

### Ionomer-Fluorophore Blending

**Figure 2:**
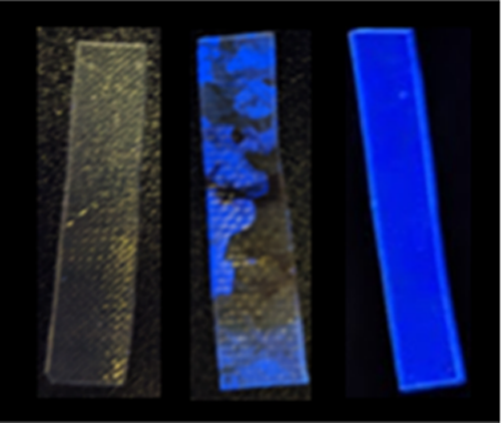
Digital photographs of the PEMA film illuminated under short-wave UV light. Films were prepared via solution blending without a fluorophore (left), with a non-ionic DPP precursor (center), and with [DPP][I] (right).

PEMA blends were fabricated via solution blending with toluene/isopropanol solvent mixtures. As a first iteration, a solution-processed PEMA film was processed with toluene and isopropanol without a fluorophore, casted into an aluminum foil mold, dried, and compressed into thin films. Visual analysis using a short-wave UV lamp showed little to no fluorescence from this polymer film alone (Figure 2). PEMA-fluorophore composite samples prepared with non-ionic DPP, however, demonstrated distinct incompatibilities with the polymer matrix. Notably, uniformity is achieved by simple ionization of this probe. Based on its uniform distribution in the ionomer, [DPP][I] was determined to be a suitable probe system for further spectroscopic analyses.

**Figure 3:**
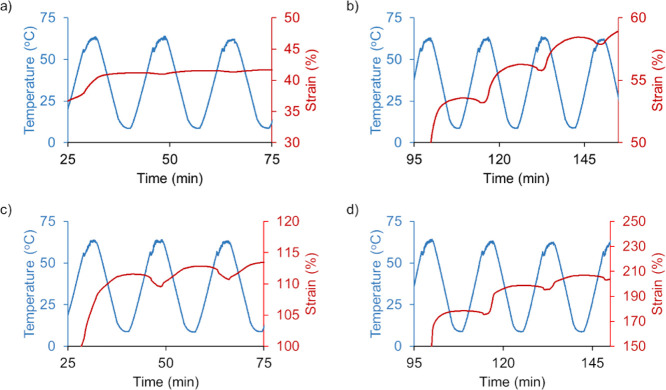
TWSME of PEMA: tensile actuation strain with 1.75 MPa tensile stress for (a) PEMA and (b) PEMA–PI composite and tensile actuation strain with 2.75 MPa tensile stress for (c) PEMA and (d) PEMA–[DPP][I] composite.

Thermomechanical effects of fluorophore incorporation were also evaluated. The storage modulus increased in GUMBOS-PEMA film samples (Figure SI-1a). Figure SI-1b is a display of tan(δ) curves of PEMA and PEMA–[DPP][I] films, which also exhibits a slight decrease in glass transition temperature. Analysis of *T*_g_ values from Table 1 indicates a slight decrease to 67.3 °C in composite films from 70.6 °C of the pure PEMA film. This indicates a change in the ratio of elastic and viscous properties of the polymer. To determine the effect of this change in the moduli ratio on shape memory abilities of PEMA, comparative two-way shape memory effect (TWSME) experiments were performed on PEMA and PEMA–[DPP][I] films (Figure 3). Table 1 lists average glass transition temperatures (*T*_g_), elongation upon cooling (EUC), and contraction upon heating (CUH) of respective 2WSME experiments at two tensile actuation stresses evaluated (1.75 and 2.75 MPa).

**Table 1:** Thermomechanical Properties of Ionomer and Composite Material

Film	*T*_g_ (°C, SD)	stress (MPa)	CUH (%)	EUC (%)
PEMA	70.6 (0.44)	1.75	0.22 (0.02)	0.36 (0.09)
		2.75	0.47 (0.09)	1.67 (0.16)
PEMA–[DPP][I]	67.3 (1.24)	1.75	2.00 (0.10)	2.10 (0.46)
		2.75	3.23 (0.57)	6.10 (0.52)

Figure 3a,d is the display of 2WSME experiments of the PEMA and GUMBOS-PEMA composite. In order to program each film sample, the local temperature was set to 65 °C. For 2WSME cycles, temperatures were fluctuated from 10 to 65 °C using 10 °C/min heating and cooling rates with 3 min isothermal intervals. These investigations revealed that incorporation of [DPP][I] into the PEMA matrix enhanced its 2WSME, as it has larger actuation strains than PEMA. Overall, the PEMA–[DPP][I] sample maintained approximately 2.00% elongation and contraction upon cooling and heating at a lower stress of 1.75 MPa, and both CUH and EUC increased when a higher stress of 2.75 MPa was applied. A film sample without a fluorophore, however, displayed a comparatively smaller actuation at both low and high stress loadings under these conditions, indicating that [DPP][I] GUMBOS increases the 2WSME at the temperature range investigated than the parent ionomer. With the observed 2WSME, the continued presence of the ionic clusters within the PEMA–[DPP][I] system should provide a suitable CTH mechanism to close punctures by themselves due to CUH.

### PEMA-GUMBOS Film Properties

**Figure 4:**
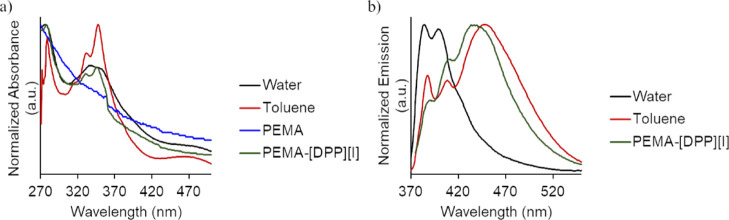
(a) UV–vis absorbance spectra of [DPP][I] in water (black line), [DPP][I] in toluene (red line), the PEMA film alone (blue line), and the PEMA–[DPP][I] film (green line) and (b) fluorescence emission (lexc = 345 nm) [DPP][I] in water (black line), [DPP][I] in toluene (red line), and the PEMA–[DPP][I] film (green line).

Optical properties of GUMBOS-incorporated films were also examined. Figure 4a depicts comparative examples for normalized UV–vis absorbance spectra for [DPP][I] in solution along with PEMA and PEMA–[DPP][I] films. Comparison of absorbance and emission spectra of [DPP][I] in different solvents demonstrates potential solvatochromic effects of this fluorophore in media of different polarities (Figure 4a,b). As shown in Figure 4a, PEMA demonstrates a gradual increase in absorbance from 500 to 270 nm, with no distinguishable peaks. Samples loaded with [DPP][I], however, display the peak characteristic of the pyrenyl rings present in the fluorescent [DPP][I]-GUMBOS probe and is similar in profile to [DPP][I] in toluene. Normalized emission spectra of [DPP][I] in water and toluene solutions, along with spectra of [DPP][I] incorporated into PEMA films, are also shown in Figure 4b. As shown, PEMA–[DPP][I] maintains optical characteristics similar to [DPP][I] in toluene in emission spectra with slight differences in peak intensities. In this regard, emission spectra were measured using an excitation wavelength of 345 nm, which is green in color and an emission wavelength in the blue region. For these reasons, we employed UV light for digital photographs of damaged and CTH areas and blue and green filters for fluorescence microscopic analyses.

**Figure 5:**
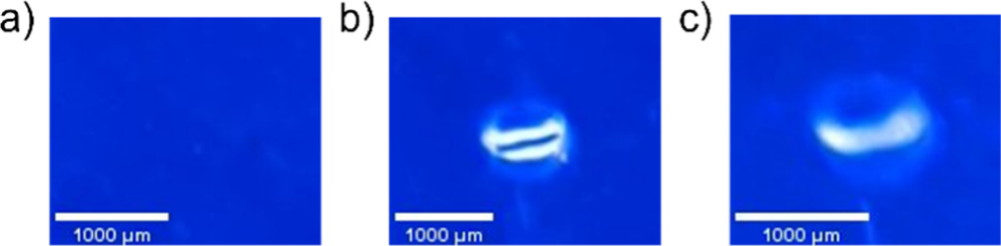
Digital photographs of PEMA–[DPP][I] (a) without damage, (b) with a heat-damaged puncture, and (c) healed puncture after CTH.

A series of digital photographs of GUMBOS-PEMA films using short-wavelength UV illumination at various stages of damage are presented in Figure 5. Figure 5a shows a uniform composite film with no damage, and Figure 5b is a photograph of the same composite film with a puncture site employing the heat-damage procedure outlined in the methods section. As shown, this puncture site has a much brighter visual illumination around edges of the puncture site as compared to the rest of the polymer film. This brightness is also maintained in Figure 5c, when CTH has been performed via external heating. In order to rule out if this illumination is a result of reflection from the UV light, fluorescence microscopy was also performed.

**Figure 6:**
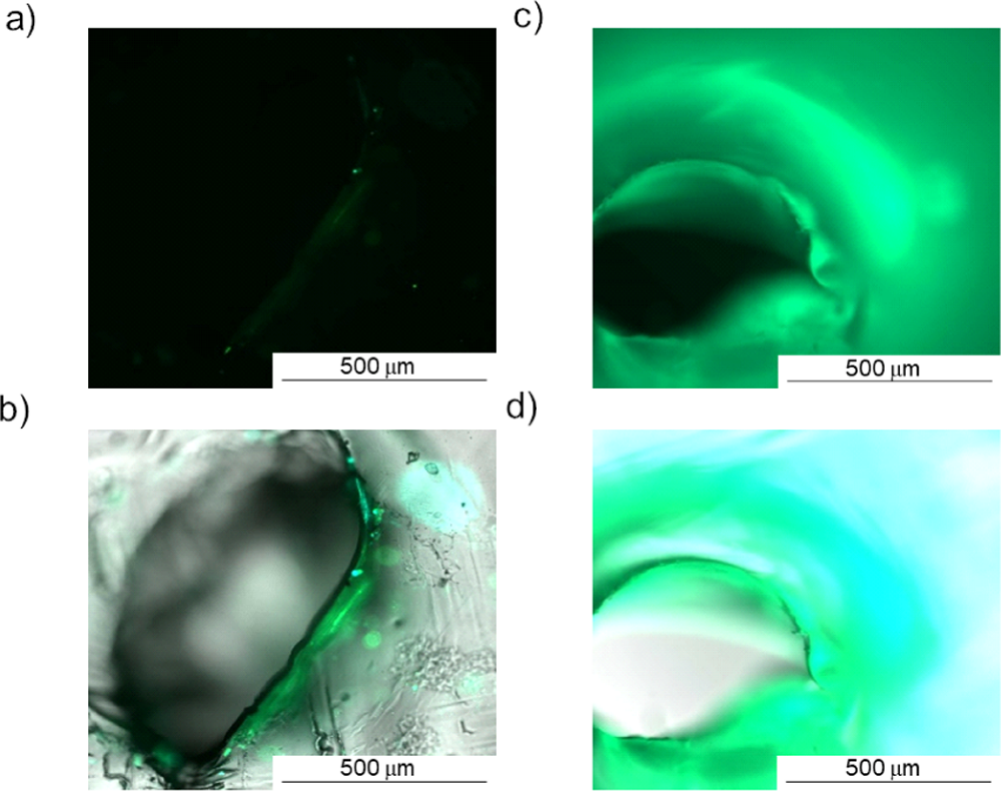
Fluorescence microscope images of puncture sites of PEMA without a fluorophore (a) green filter and (b) blue, green, and bright field image overlays compared to the composite film with (c) green filter only and (d) with overlaid images of blue, green, and bright field filters.

A puncture site of the PEMA film without the GUMBOS probe in Figure 6a shows little to no illumination using a green filter with only slight reflection of green and blue light filters in the brightfield overlay of Figure 6b. When using a punctured PEMA–[DPP][I] film, however, this site is substantially clarified (Figure 6c). Overlaid images of blue, green, and bright field filters (Figure 6b,d) display clear distinctions between edges of the damaged site in PEMA and PEMA–[DPP][I] composites. Although some reflection is observed around the puncture site, Figure 6b,d illustrates that the fluorophore composite maintains an enhanced distinction of green and blue contrast with the brightfield overlay. Heat-punctured areas display deeper green hues that slightly fade to blue and further to light blue-white, where damage did not occur, which could indicate a potential visual use of this fluorophore to detect damage.

**Figure 7:**
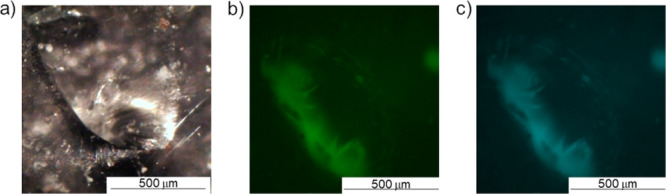
Micrographs of the healed puncture site using (a) optical microscope and fluorescence microscope images of puncture sites, (b) green filter, and (c) green and blue filter overlay.

Fluorescence microscopy was also employed to observe if visual monitoring of healing of CTH sites may be achieved. Figure 7a is a representative digital microscope image of a PEMA–[DPP][I] composite film. It is apparent that the puncture site has fully closed, and further fluorescence microscopic images were taken (Figure 7b,c). Figure 7b shows a sample of this healed composite under a green filter, with substantial illumination around the edges in the healing site. Figure 7c was taken as an overlay of blue and green filters to determine if any variations around this site could be achieved. It was apparent that this overlay did not show distinctions with blue and green filters. Although this specific strategy may not provide sufficient support for a scientific visual detection method at this stage of study, future work will be employed to investigate other optically active, ionic probes for a visual mode of healing progress and damage detection.

### Puncture Healing: Composite CTH and Ionic Interaction

Experiments to promote CTH were performed in a manner that facilitated healing through external heating events on both sides of the polymer thin films. Visible puncture site closure was achieved via shape recovery during these healing events. It has been shown that ionomers undergo an autonomous self-healing process in damage recovery.^27,60^ During this process, a reassembly of the ionic clusters within the ionomer may orient to not only close a damaged site but heal the site as well. We propose that emission variances observed could be a result of the various changes in local ionic components within the healing site. To further support our hypothesis that the local ionic component changes over time, we also performed differential scanning calorimetric studies to observe and compare the effect to undamaged composite film samples.

Differential scanning calorimetry (DSC) was employed to further investigate molecular phase interactions among crystalline and amorphous segments after healing of the ionomer films.^29,61^ Control samples, such as an undamaged PEMA–[DPP][I] film and a film that was exposed to 5 MPa tensile stress using DMA elongation, were also analyzed. Figure SI-2 are representative endotherms for all samples. Thermal properties of each measurement are summarized in Table 2 mentioned below.

**Table 2:** Thermal Properties Determined by DSC Analysis of PEMA and Composite Films

Damage	PEMA	PEMA–[DPP][I]
polyethylene (PE, *T*_m_, °C)	90.22 (0.05)	91.95 (0.63)
crystallinity (*X*_c_, %)	11.25 (0.41)	9.66 (2.00)
methacrylic acid (MA, *T*_m_, °C)	44.95 (2.92)	51.47 (2.16)
recrystallization (PE, *T*_c_, °C)	55.35 (0.61)	56.93 (1.22)

As determined from PEMA films with no damage, characteristic melting peaks for segments associated with polyethylene (PE) units were observed at 90.22 °C.^28,29^ With PEMA only, the order to disorder transition peak associated with the methacryclic acid (MA) units was recorded at 44.95 °C, and the recrystallization peak was observed at 55.35 °C. For PEMA–[DPP][I], melting and recrystallization peaks associated with PE remain at the same temperatures in undamaged samples. However, the endotherm peak associated with secondary crystallites, or methacrylic acid units (MA),^24^ increased to 51.47 °C, which may be the result of crystallite annealing within the composite.^62,63^

Distinguishable transitions from order to disorder MA and PE segments were observed upon analysis of CTH samples, and results are recorded in Table 3. Samples analyzed 1 day after CTH were determined to have slightly lower recrystallization temperatures, and *T*_m_ associated with MA decreased to 42.29 °C. Notably, an appearance of a third endotherm peak was observed at 67.38 °C in these samples. In endotherms corresponding to CTH samples analyzed 11 days after healing, melting points of MA units also decreased to 45.35 °C with a marked decrease in intensity. Additionally, these samples did not display a distinguishable third endotherm peak, and peaks associated with PE segments displayed significantly decreased melting temperatures to 85.35 °C with a shoulder at approximately 91.1 °C and increased crystallinity.

**Table 3:** Thermal Properties of CTH Composite Films Determined by DSC Analysis

damage	1 day^a^	11 days^b^	5 MPa stress^c^
PE (*T*_m_, °C)	91.1 (0.08)	85.35 (0.49)	91.05 (0.76)
PE (*X*_c_, %)	8.00 (2.00)	12.10 (1.00)	12.14 (4.58)
MA (*T*_m_, °C)	42.29 (3.18)	45.35 (3.29)	48.36 (1.24)
PE (*T*_c_, °C)	58.87 (0.85)	56.30 (0.23)	57.94 (2.20)

a Third endotherm observed at 67.38 °C (±0.76).

b Peak shoulder observed at 91.1 °C (±0.00).

c Broad endotherm observed at 65.46 °C (±5.00).

We hypothesize that appearance of the third endotherm peak in CTH samples analyzed 1 day after healing and decreased PE melting temperature in 11 days post-healing samples indicate annealing of ionic units at the puncture site and co-relaxation of the ionic content during melting and recrystallization with PE at CTH sites at different time points.^24,28,29,63,64^ To further investigate this hypothesis for increase in localized ionic units independent of damage and time, we performed additional DSC analysis with polymer composite sample segments that were exposed to 5 MPa tensile stress.^65^ In this regard, a decrease in temperature associated with MA segments was also observed along with an increase in percent crystallinity. Additionally, the fluorescence emission values for both CTH samples were recorded before performing DSC studies. These ratios are presented in Figure SI-3 along with other recorded emission values for further CTH studies in the following sections.

### Evaluating Mechanical Properties after CTH with GUMBOS Probe

In this study, mechanical properties, such as Young’s modulus (YM) and stress response values measured at 10% strain, were evaluated, and averages of these values are shown in Table 4. In general, an increasing trend in YM was observed over time and 16 days after CTH samples were determined to have similar values relative to samples that were not damaged.

**Table 4:** Mechanical Responses of PEMA–[DPP][I] Films after CTH

post-CTH	YM (MPa, SD)	stress at 10% strain (MPa, SD)
5 min	141 (18)	7.7 (0.9)
1 h	153 (27)	10 (2)
1 day	194 (48)	9.5 (0.5)
3 days	186 (21)	10.1 (0.6)
8 days	204 (61)	8.9 (0.6)
16 days	244 (70)	12 (1)
no damage	241 (91)	12 (2)

**Figure 8:**
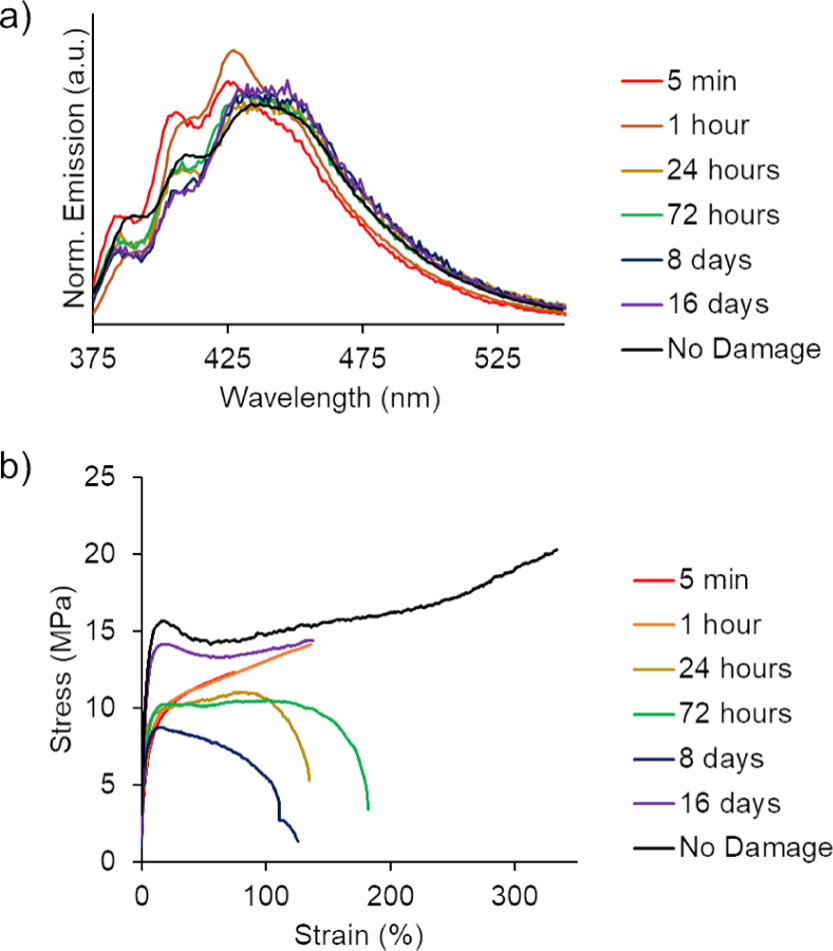
Emission intensity peaks (*I*_1_ and *I*_2_) are defined as the intensity of the first peak, over the intensity of the second peak.

**Figure 9:**
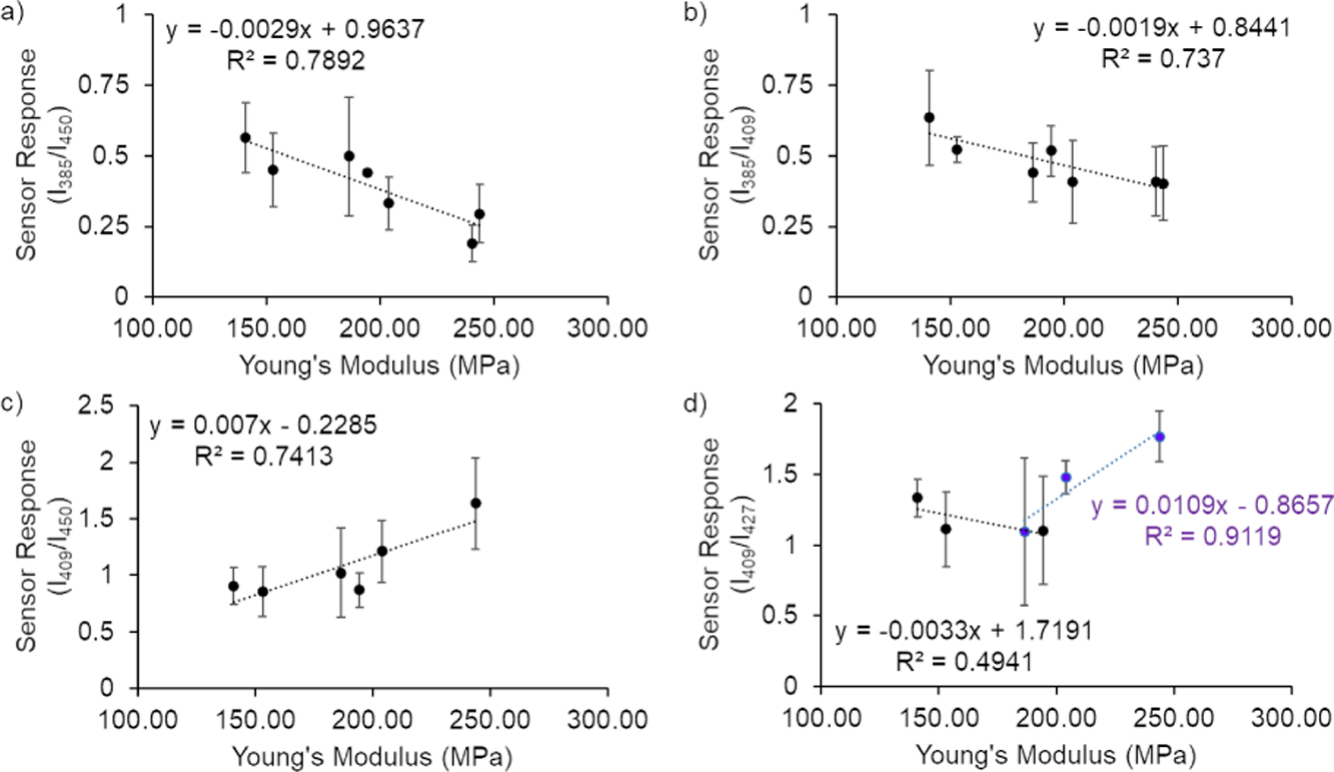
Sensor responses vs YM with undamaged data points (a) *I*_385_/*I*_409_ and (b) *I*_385_/*I*_450_ and without undamaged data points (c) *I*_409_/*I*_450_ and (d) *I*_409_/*I*_427_.

Because visual analysis was inconclusive with providing sufficient information regarding time since damage healing, fluorescence measurements were recorded. As shown in Figure 8a, the emission peaks of the fluorescence spectra changed over time, for example, 5 min after CTH demonstrated a higher relative intensity of peaks 385 and 409. In contrast, samples analyzed after 16 days CTH demonstrated an emission profile similar to that of an undamaged composite sample. As a result, two methods were employed to investigate if a correlation could be made between emission intensity ratios and mechanical properties from the stress–strain profiles obtained at each time point (Figure 8b). In the first method of emission ratio analysis, an attempt was made to directly relate emission ratios with a mechanical property, such as YM. The results of this comparison are shown in Figure 9.

In order to investigate if relative emission ratios were correlated to time since CTH or other mechanical properties, fluorescence emission spectra of PEMA–[DPP][I] composites were obtained immediately before tensile testing at various times after CTH.
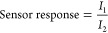
1

Different ratios considering different emission peaks of [DPP][I] GUMBOS have been evaluated in this study. From all the ratios, some such as *I*_385_/*I*_450_ showed a linear relationship between the sensor responses and YM of the PEMA before and after damage occurred (Figure 9). Sensor responses were then calculated by dividing normalized peak values at 385, 409, 427, and 450 nm of UV light as described in eq 2 mentioned above. Each sensor response was plotted against mechanical information to determine if there was a direct relationship between the optical sensor response and mechanical property. Figure SI-3 shows graphical representation of sensor responses explored.

Upon examination of all sensor responses with YM values, the emergence of two linear trends was observed when employing all sample information, including undamaged samples, and when employing YM values as observed in Figure 9a,b. In Figure 9a, larger standard deviations in responses are observed below 190 MPa. These results indicate that this GUMBOS probe could be used to provide YM information above this value in film samples when *I*_385_/*I*_450_ values are employed for analysis. When *I*_409_/*I*_450_ responses from only damaged film samples were compared to YM, another linear trend was observed (Figure 9c). Interestingly, *I*_409_/*I*_427_ responses demonstrated low linearity at YM values below 200 MPa but were determined to have a linear relationship with YM values greater than this. The linearity ranges within YM values of these sensor responses could indicate an optical relationship between recovered mechanical properties of a damage-healed fluorophore-doped ionomer. Attempted correlations to stress recorded at 10% strain values resulted in poor linear trendlines, as larger variations in these values were observed among the CTH time points studied (Table 3). Thus, discriminant analysis was employed as the second method of emission ratio analysis as a result of relatively large standard deviation sensor responses and low linear correlation for these values.

**Figure 10:**
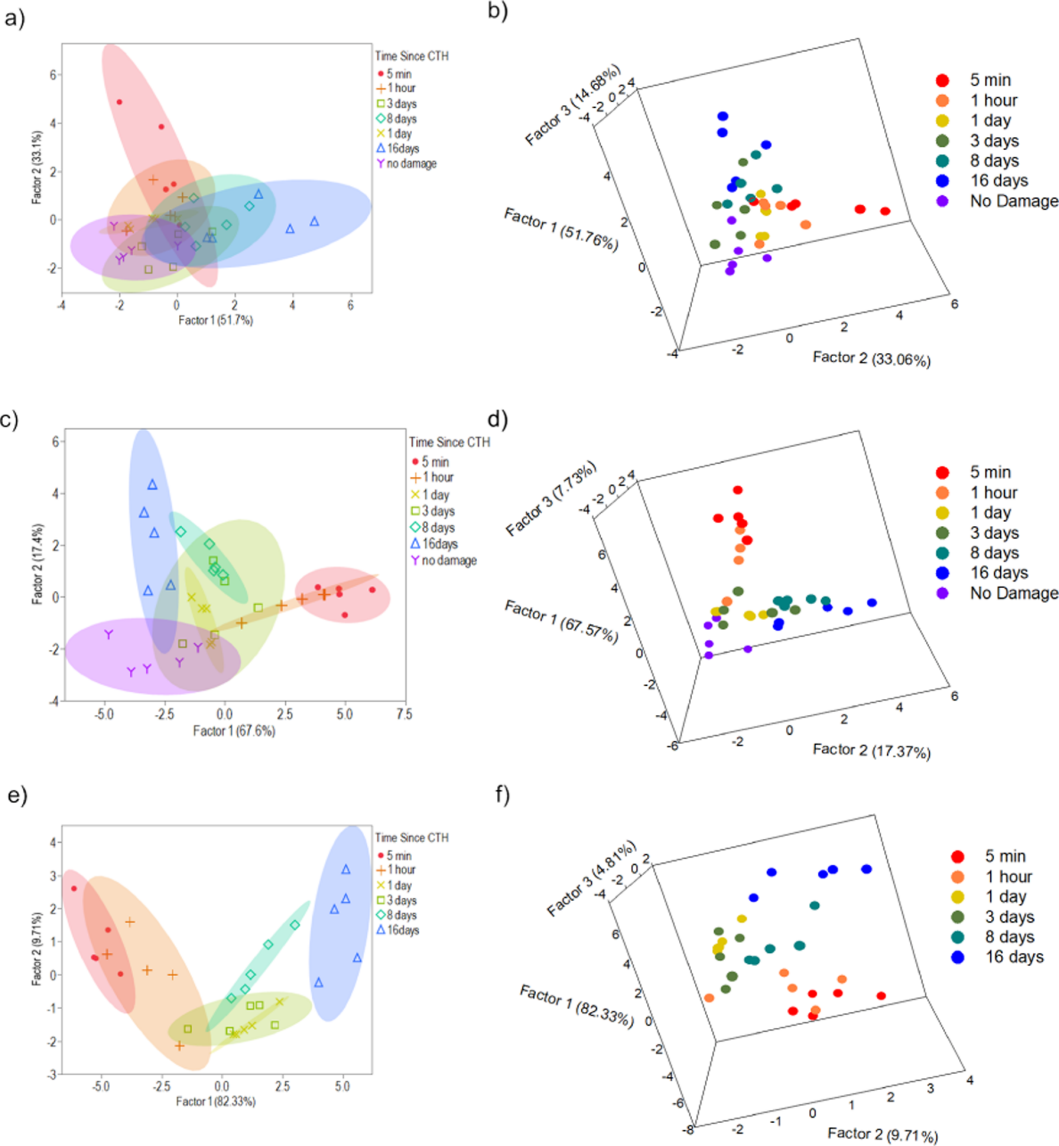
QDA plots employing (a) all sensor responses and (b) corresponding three-dimensional plot (35 measurements), (c) employing all sensor responses and stress values recorded at 10% strain and (d) corresponding three-dimensional plot (35 measurements), and (e) employing all sensor responses and stress recorded at 10% strain without undamaged film responses and (f) corresponding three-dimensional plot (30 measurements). Ellipses represent a 95% confidence level for all 2D plots.

Quadratic discriminant analysis (QDA) with cross-validation was employed using sensor responses as input variables to construct predictive models.^56,57,66−68^ This statistical method uses input variables to reduce dimensionality of datasets and for discrimination between designated categories of these variables. In this regard, all sensor responses for 38 samples were employed using QDA as shown in Figure 10a. In this model, significant overlap of ellipses that represent 95% confidence levels was observed. However, 90% accuracy was obtained among these samples, where data points after CTH occurred were misclassified as follows: one sample of 16 days was misclassified as 8 days, one sample of 3 days was misclassified as 8 days, and one sample of 5 min was misclassified as 1 h. Although the accuracy of this model was relatively high, poor visibility of the accuracy could be a result of three-dimensional (3D) effects not easily observed in a two-dimensional (2D) model. For this reason, a 3D plot of this QDA model is shown in Figure 9b. Although there is a more distinguishable separation of datasets in 3D, overlap of these categories remains significant.

Notably, when YM values were employed as the associated mechanical property input variable, poor QDA discrimination was achieved. As a result of this overlap, stress values recorded at 10% strain were employed as a mechanical property to assist in QDA model construction (Figure 10c). The strategy of employing stress values recorded at 10% strain along with sensor responses as input variables resulted in 94.29% accuracy among the 35 samples. In this case, only two data points were misclassified, both of which were among 3 days after the CTH dataset, where one point was classified as an undamaged sample, and one point was misclassified as 16 days after the CTH sample. Visually, this model displays ellipses that are more concise and indicates a better relative degree of 2D separation among categorized samples versus the previous model with larger category separation displayed in a 3D plot (Figure 10d).

Finally, a third model was constructed to determine if predictive model construction could be viable among only samples that have been damaged with sensor responses and stress values recorded at 10% strain as input variables for QDA with cross-validation (Figure 10e). In this regard, ellipses that represent 95% confidence levels display an even greater relative degree of separation. As a result of this analysis, 96.67% accuracy was achieved among 30 samples, where one 3 days sample after the CTH data point was misclassified under the 16 days category. In this model of statistical analysis, improved separation between categorized samples is more apparent in both 2D and 3D (Figure 10e,f) formats. As a result, this GUMBOS probe displays properties of a potential probe to monitor recovered properties of damaged-then-healed ionomer composites of PEMA, which could translate to quality monitoring of desired applications.

## Conclusions

In conclusion, this work provides a study of the blending effects of non-ionic and ionic fluorophores in sodium-neutralized poly(ethylene-*co*-methacrylic) acid. By simple transformation to ionic [DPP][I] GUMBOS, this fluorescent probe was determined to achieve uniformity and compatibility with this polymer matrix. Based on this uniformity, thermal and optical characterization studies were performed, along with confirmation that 2WSME capabilities were maintained. Puncture-healing studies were also performed and analyzed through DSC studies for investigation of properties after CTH phenomena of these fluorophore-loaded films. Emission analysis of these closed-then-healed films at various time points post-healing resulted in observed ratiometric trends of several intensity ratios with YM in good linear relationships. Three highly accurate models constructed by QDA with cross-validation were also explored and discussed. Finally, while results obtained are very encouraging, we note that the dye combination chosen here was the only one explored. Other dye combinations may prove even more fruitful for detection and monitoring of wound healing. Thus, further development of GUMBOS may be even more useful for comprehensive monitoring of damage healing of ionomers and potential development of better predictive sensors.

## Materials and Methods

Unless otherwise noted, all chemicals and solvents were obtained from commercial sources and used as received. Dimethylformamide (DMF), methanol (MeOH), dichloromethane (DCM), toluene, and isopropanol (^*i*^PrOH) were purchased from VWR International. Iodomethane (MeI, 99.9% purity) was purchased from Sigma-Aldrich (St. Louis, MO). PEMA pellets (Surlyn 8940) were purchased from Dupont. Quartz glass slides, manufactured by Chemglass, were purchased from VWR (Batavia, IL).

### Ionomer Blending and Thermomechanical Experiments

Composites were fabricated using a solution-blending procedure. PEMA was heated between 100 and 110 °C in a 3:1 solution of toluene and isopropanol (3 g in 40 mL) until the ionomer was visibly dissolved. A small aliquot of fluorophore solution (600 μL, 1 M) was added and stirred while heating for 1 h at approximately 100 °C. Composites were then poured into aluminum foil molds, and the solvent was allowed to evaporate at room temperature for 48 h in ventilated hoods. All composites were hot-pressed at approximately 150 °C to form thin films. DSC experiments were performed in duplicate using 3–5 mg samples with a DSC Q100 (TA Instruments, New Castle, NJ) using heating and cooling rates of 10.00 °C/min from −40 to 150 °C. Percent crystallinity (*X*_c_) of PE segments was calculated using eq 2 mentioned below
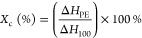
2where Δ*H*_PE_ is the area under the designated PE peak calculated using TA Universal Analysis software, version 4.5A, and Δ*H*_100_ is a constant value for 100% PE crystallinity of 293 J/g.^69,70^

A DMA Q800 (TA Instruments, New Castle, NJ) was employed for dynamic mechanical analysis measurements, such as the tan delta [tan(δ)], 2WSME, and stress–strain curves. Heating and cooling rates were maintained at 10.00 °C per minute, with 1 Hz, and a preload force of 0.001 N for tan(δ) and 2WSME experiments. Five replicates of stress–strain curves were performed at room temperature at allotted times after CTH using a strain rate of 10.00% per minute.

Composite films received punctures with dimensions of 10.00 × 4.75 × 0.25 mm^3^. To achieve this, a 20-gauge needle (BD Scientific) was placed in an oven set to 130 °C for 5 min.^29,30,64,65^ Needles were used to completely puncture thin films, and CTH was initiated via external heat exposure at 50 °C to promote puncture site closure.^22,24^ Both sides of the films were heated for 2.5 min to produce a total healing time of 5 min. Samples were covered in aluminum foil and stored at ambient temperature until fluorescence measurements and tensile testing were performed at designated times.

### Optical Measurements

Absorbance measurements of films were obtained using a UV-3101PC spectrophotometer (Shimadzu, Columbia, MD). Fluorescence emission spectra were obtained using a HORIBA Spex Fluorolog-3-spectrofluorometer (model FL3-22TAU3; Jobin Yvon, Edison, NJ). All optical measurements were performed with a slit width of 5 nm. Films were mounted onto quartz glass slides for spectrophotometric measurements. Fluorescence microscopy images were obtained using a Leica DM6B upright microscope equipped with a Hamamatsu sCMOS camera, Leica DFC450 color CCD, and Spectra X LED light engine and employing green, blue, and bright field filters.

## Supporting Information Available

Additional experimental information including synthesis and characterization of probes, thermal characterization of composites, and sensor responses after CTH events (PDF)

## Abbreviations

[DPP][I]: 1-methyl-4,6-dipyrenylpyrimidinium iodide
DPP: 4,6-dipyrenylpyrimidine
PE: polyethylene
MA: methacrylic acid
PEMA: poly(ethylene-*co*-methacrylic acid)
SD: standard deviation
CTH: close-then-heal
^*i*^PrOH: isopropanol
DMF: dimethylformamide
MeOH: methanol
DCM: dichloromethane
DSC: differential scanning calorimetry
DMA: dynamic mechanical analysis
YM: Young’s modulus
EUC: elongation upon cooling
CUH: contraction under heating
QDA: quadratic discriminant analysis
