# Supplementary material for: Fluorescent Ionic Probe for Determination of Mechanical Properties of Healed Poly(ethylene-co-methacrylic acid) Ionomer Films
Source: ACS Appl Polym Mater. 2022 Feb 2;4(2):832–41. doi: 10.1021/acsapm.1c01325 (PMC8845041; doi:10.1021/acsapm.1c01325)
Supplement: Supplementary file 1 — ap1c01325_si_001.pdf [file ap1c01325_si_001.pdf]

Supporting Information:
Fluorescent Ionic Probe for Determination of Mechanical Properties of Healed Poly(ethylene-*co*-methacrylic acid) Ionomer Films

Caitlan E. Ayala[a], Rocío L. Pérez[a,b], John K. Mathaga[a], Aanesa Watson[c], Tristan E. Evans[a], and Isiah M. Warner[a*]

[a] Department of Chemistry, Louisiana State University, Baton Rouge, Louisiana 70803, USA.
[b] Department of Chemistry and Biochemistry, Georgia Southern University, Statesboro, GA, 30458, USA.

[c] Department of Chemistry, Fort Valley State University, Fort Valley, Georgia 31030, USA.
*Corresponding Author: Caitlan Ayala, E-mail: ayala1@lsu.edu; Isiah M. Warner, E-mail: iwarner@lsu.edu; Fax: +1 225-578-3458; Tel: +1 225-578-2829.

Synthesis and characterization of fluorophores:

Non-ionic 4,6-DPP was synthesized according to previously reported procedures with slight modifications .[53-54] Pyrene-1-boronic acid (7.20 mmol, 1.80 g), 4,6-dichloropyrimidine (3.0 mmol (447 mg), bis(triphenylphosphine)palladium chloride (0.15 mmol, 105 mg), and potassium carbonate solution (2.0 mol in 50 ml) with 120 mL of 1,4-dioxane were stirred for 24 hrs at 90 °C in a nitrogen atmosphere. After cooling to room temperature, the mixture was poured into ice water, and resultant solid was filtered. Crude filtrate was washed several times with 300 mL brine solution and freeze-dried. Crude solids were then purified via silica gel chromatography with 60% chloroform in hexane. Collected fractions were concentrated by rotary evaporation and further purified by reprecipitation several times to obtain a brownish yellow solid.

ESI-HRMS m/z = $(M+H)^+$ expected = 481.1699, found = 481.1691 for $(C_{36}H_{20}N_2+H)^+$; ^{1}H NMR ($CDCl_3$, 500 MHz, ppm): δ 9.73 (d, J = 1.0 Hz, 1H), 8.71 (d, J = 9.20 Hz, 2H), 8.35 (dd, J = 13.65, Hz, 4H), 8.27 (t, J = 14.25 Hz, 5H), 8.22-8.15(m, 6H), 8.08 (t, J = 7.58 Hz, 2H); ^{13}C ($CDCl_3$, 125 MHz, ppm): δ 167.06, 159.12, 132.82, 132.54, 131.34, 130.81, 128.93, 128.83, 128.79, 127.66, 127.34, 126.35, 125.96,125.65, 125.16, 125.00, 124.71, 124.16, 123.92.

Ionic [DPP][I] was synthesized using a traditional methylation strategy.[55] In brief, 4,6-DPP (215 mg) was dissolved in 20 mL DMF in a pressure vessel. Excess iodomethane (3 mL) was added, and the pressure vessel was sealed. The reaction mixture was maintained between 90 and 100 °C for 72 hrs, cooled to room temperature, and DMF was removed via rotary evaporation. Crude product was washed several times with distilled water to remove residual DMF. Crude material was then purified via flash column chromatography (0.1% MeOH in DCM). Pure fractions were collected and concentrated by rotary evaporation, washed several times with diethyl ether, and dried to provide an orange powder.

ESI-HRMS m/z = $(M)^+$ expected = 495.1856, found = 495.1870 for $(C37H23N2)^+$; ^{1}H NMR (DMSO-D6, 500 MHz, ppm): δ 10.23 (s, 1H), 9.04-9.00 (m, 2H), 8.53-8.29 (m, 12H), 8.34 (d, J = 8.9Hz, 1H), 8.26-8.20 (m, 3H), 4.05 (s, 3H). ^{13}C (DMSO-D6, 125 MHz, ppm): δ 170.94, 160.40, 154.67, 133.36, 132.66, 130.20, 130.14, 129.76, 129.67, 129.62, 129.31, 129.29, 128.90, 128.76, 128.37, 127.86, 126.74, 126.64, 126.61, 126.56, 126.48, 126.19, 126.07, 125.69, 124.70, 124.53, 123.61, 123.45, 123.24, 123.05, 123.00, 122.87, 122.79, 62.26.

Thermomechanical and Optical Characterization of PEMA and Composite Films

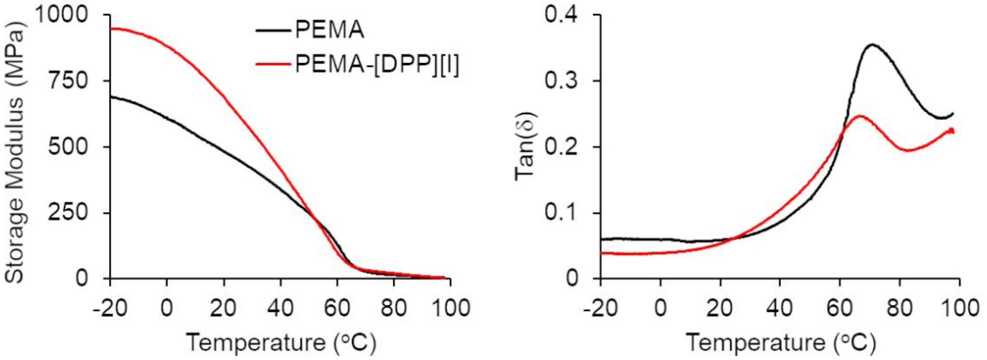

Figure SI-1: a) Storage modulus and b) Tan(δ) versus temperature curves of PEMA films (black lines) and PEMA-[DPP][I] films (red lines).

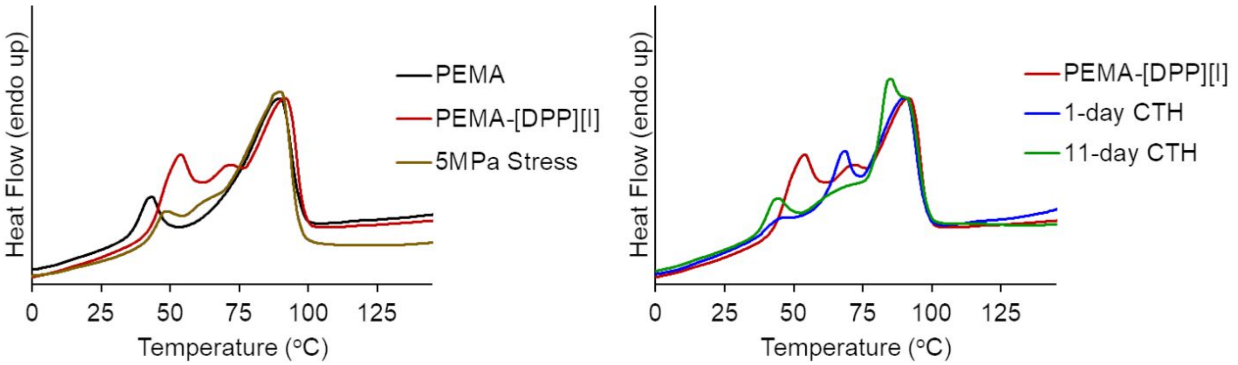

Figure SI-2. DSC heating profiles for PEMA and PEMA-GUMBOS composites.

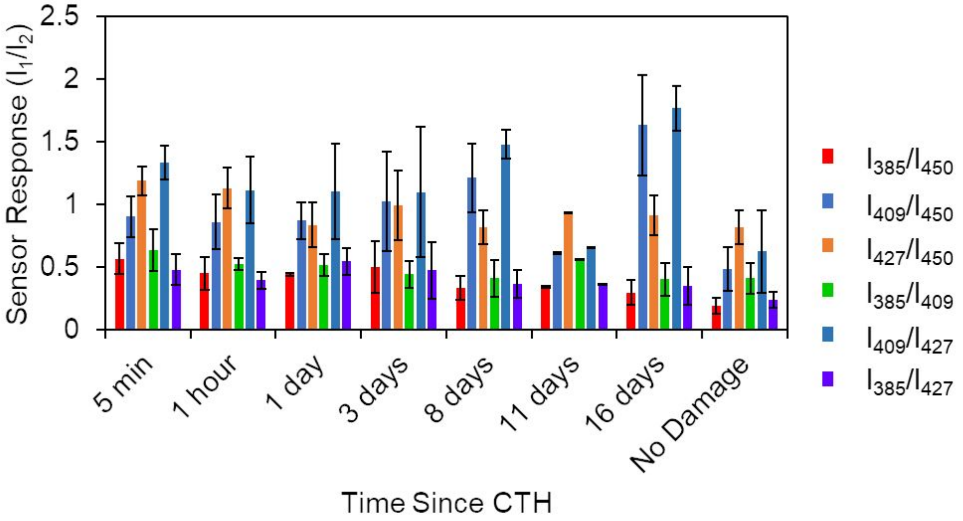

Figure SI-3. Sensor responses after CTH events.
